# Combination chemotherapy for choroidal melanoma: *ex vivo* sensitivity to treosulfan with gemcitabine or Cytosine arabinoside

**DOI:** 10.1038/sj.bjc.6690237

**Published:** 1999-03

**Authors:** M H Neale, N Myatt, I A Cree, C M Kurbacher, A J E Foss, J L Hungerford, P N Plowman

**Affiliations:** Institute of Ophthalmology, Department of Pathology, Bath Street, London, EC1V 9EL, UK; Labor fur Chemosensitivitatestungen, Universitats – Frauenklinik, Kerpener Strasse 34, Cologne, 50931, Germany; Department of Ophthalmology, Queens Medical Centre, Nottingham, NG7 2UH, UK; Moorfields Eye Hospital, City Road, London, EC1V 2PD, UK; Department of Radiotherapy and Oncology, St Bartholomew's Hospital, West Smithfield, London, EC1A 7BE, UK

**Keywords:** chemosensitivity, melanoma, eye, choroid, ATP, DNA repair, apoptosis, anti-neoplastic agents, combination

## Abstract

Treatment of choroidal melanoma by chemotherapy is usually unsuccessful, with response rates of less than 1% reported for dacarbazine (DTIC)-containing regimens which show 20% or more response rates in skin melanoma. Recently, we reported the activity of several cytotoxic agents against primary choroidal melanoma in an ATP-based tumour chemosensitivity assay (ATP-TCA). In this study, we have used the same method to examine the sensitivity of choroidal melanoma to combinations suggested by our earlier study. Tumour material from 36 enucleated eyes was tested against a battery of single agents and combinations which showed some activity in the previous study. The combination of treosulfan with gemcitabine or cytosine arabinoside showed consistent activity in 70% and 86% of cases, respectively. Paclitaxel was also active, particularly in combination with treosulfan (47%) or mitoxantrone (33%). Addition of paclitaxel to the combination of treosulfan + cytosine analogue added little increased sensitivity. For treosulfan + cytosine arabinoside, further sequence and timing experiments showed that simultaneous administration gave the greatest suppression, with minor loss of inhibition if the cytosine analogue was given 24 h after the treosulfan. Administration of cytosine analogue 24 h before treosulfan produced considerably less inhibition at any concentration. While we have so far been unable to study metastatic tumour from choroidal melanoma patients, the combination of treosulfan with gemcitabine or cytosine arabinoside shows activity *ex vivo* against primary tumour tissue. Clinical trials are in progress. © 1999 Cancer Research Campaign


*British Journal of Cancer* (1999) **79**(9/10), 1487–1493
© 1999 Cancer Research Campaign
Article no. bjoc.1998.0237

### Combination chemotherapy for choroidal melanoma: *ex vivo* sensitivity to treosulfan with gemcitabine or Cytosine arabinoside

**MH Neale[1], N Myatt[1], IA Cree[1], CM Kurbacher[2], AJE Foss[3], JL Hungerford[4] and PN Plowman[5]**

[1]Department of Pathology, Institute of Ophthalmology, University College London, Bath Street, London EC1V 9EL, UK; [2]Labor fur Chemosensitivitatestungen, Universitats – Frauenklinik, University of Cologne Medical Centre, Kerpener Strasse 34, Cologne 50931, Germany; [3]Department of Ophthalmology, Queens Medical Centre, Nottingham NG7 2UH; [4]Moorfields Eye Hospital, City Road, London EC1V 2PD; and [5]Department of Radiotherapy and Oncology, St Bartholomew's Hospital, West Smithfield, London EC1A 7BE, UK

**Summary** Treatment of choroidal melanoma by chemotherapy is usually unsuccessful, with response rates of less than 1% reported for dacarbazine (DTIC)-containing regimens which show 20% or more response rates in skin melanoma. Recently, we reported the activity of several cytotoxic agents against primary choroidal melanoma in an ATP-based tumour chemosensitivity assay (ATP-TCA). In this study, we have used the same method to examine the sensitivity of choroidal melanoma to combinations suggested by our earlier study. Tumour material from 36 enucleated eyes was tested against a battery of single agents and combinations which showed some activity in the previous study. The combination of treosulfan with gemcitabine or cytosine arabinoside showed consistent activity in 70% and 86% of cases, respectively. Paclitaxel was also active, particularly in combination with treosulfan (47%) or mitoxantrone (33%). Addition of paclitaxel to the combination of treosulfan + cytosine analogue added little increased sensitivity. For treosulfan + cytosine arabinoside, further sequence and timing experiments showed that simultaneous administration gave the greatest suppression, with minor loss of inhibition if the cytosine analogue was given 24 h after the treosulfan. Administration of cytosine analogue 24 h before treosulfan produced considerably less inhibition at any concentration. While we have so far been unable to study metastatic tumour from choroidal melanoma patients, the combination of treosulfan with gemcitabine or cytosine arabinoside shows activity *ex vivo* against primary tumour tissue. Clinical trials are in progress.

**Keywords:** chemosensitivity; melanoma; eye; choroid; ATP; DNA repair, apoptosis; anti-neoplastic agents; combination

Choroidal melanoma is a chemoresistant tumour which is fatal in about 50% cases at 10 years and has a median survival of 5 to 7 months following the development of metastases (Ravio, 1977; Albert et al, 1992; Bedikian et al, 1995). Although a high-risk group can be defined by stage (tumour size) and microvascularity (Foss et al, 1996), no adjuvant therapy is available for routine use. Primary therapy is limited to enucleation or local irradiation (Albert et al, 1992).

Treatment of metastatic choroidal melanoma with chemotherapy using regimens applied to skin melanoma has been largely unsuccessful and has been based on the premise that because the two tumours are derived from the same cell type, they may respond similarly, despite the many genetic and phenotypic differences between them (Albert et al, 1996; Chana et al, 1998). While occasional responses have been reported, there are few trial-based data from which response rates can be obtained (Albert et al, 1992). One large series reported a response rate less than 1% for systemic therapy, although chemoembolization of the liver using cisplatin-based regimens was more effective, producing responses in 36% of patients (Cantore et al, 1994; Bedikian et al, 1995). The results of these studies suggest that at least some of these tumours are partially sensitive to platinum-based therapy, although in systemic combination with other drugs, cisplatin shows little effect (Proebstle et al, 1996).

In a recent study of the chemosensitivity of uveal melanoma ex vivo (Myatt et al, 1997), we used an ATP-based luminescence assay (Hunter et al, 1993; Andreotti et al, 1995; Cree et al, 1996; Kurbacher et al, 1998) to determine the sensitivity of primary uveal melanoma to a variety of chemotherapeutic agents. We observed variable sensitivity to treosulfan, cytosine arabinoside, paclitaxel and doxorubicin and showed enhancement of the response to treosulfan by cytosine arabinoside (Myatt et al, 1997). Recently, a new derivative of cytosine arabinoside, gemcitabine, has been shown to have greater effects on solid tumours (Plunkett et al, 1995). Previous experience with gemcitabine in modulating Cisplatin activity in ovarian cancer both clinically and in vitro was encouraging (van Moorsel et al, 1997; Kurbacher et al, 1998). As a corollary, we therefore decided to explore the use of the combination of treosulfan + gemcitabine further ex vivo by chemosensitivity testing of primary uveal melanoma tumours.

*Received 20 May 1998*
*Revised 8 September 1998*
*Accepted 7 October 1998*

*Correspondence to:* IA Cree

### MATERIALS AND METHODS

### Melanomas

Material from 37 consecutive large primary intra-ocular melanomas (Table 1) was obtained under sterile conditions from fresh enucleation specimens removed consecutively at Moorfields

**Table 1** Drug concentrations used in the assay and their clinically relevant doses. For combinations, each drug was added at the TDCs to the same wells. The dose correlation represents the standard dose from which pharmacokinetic data were used to estimate the test drug concentration and is given for information only

| Drug name | Test Drug Concentration (TDC) | | Drug dose Correlation |
|---|---|---|---|
| | μ ml⁻¹ | μM | |
| Cytosine arabinoside | 2.4 | 9.87 | I.V. 100 mg $m^{-2}$ |
| Doxorubicin | 0.5 | 0.86 | I.V. 60 mg $m^{-2}$ |
| Treosulfan | 3 | 10.5 | ORAL 1 g $day^{-1}$ |
| Vincristine | 0.4 | 0.48 | I.V. 1.5 mg $m^{-2}$ |
| Vinblastine | 0.5 | 0.62 | I.V. 6.0 mg $m^{-2}$ |
| Paclitaxel | 6.8 | 7.96 | I.V. 175 mg $m^{-2}$ |
| Gemcitabine | 12.5 | 47.5 | I.V. 1250 mg $m^{-2}$ |
| Mitoxantrone | 0.3 | 0.58 | I.V. 12 mg $m^{-2}$ |

Eye Hospital or St Bartholomew's Hospital over a 9-month period. All except two were previously untreated: the exceptions had failed local ruthenium plaque or proton beam radiotherapy. Three patients had had diagnostic choroidal biopsy preceding enucleation. Enucleated eyes were examined externally for the presence of extrascleral extension (present in four cases) or previous surgery, and by transillumination using a fibre-optic light source to locate the tumour. The eye was then oriented in a steel eye-cup and sectioned posteriorly starting at the cornea to one side of the midline continuing to the same side of the optic disc. The larger calotte was placed immediately into 4% buffered formaldehyde for histopathology, while the smaller calotte without the optic disk was examined. The tumours ranged in size (largest tumour diameter) from 7–23 mm, with a median of 12 mm. Ciliary body involvement was present in four of 37 tumours, two of which appeared to originate from the ciliary body, while the remainder were restricted to the choroid. Tumour material was scraped from the calotte and placed into 10 ml of Dulbecco's Modified Eagle's Medium (DMEM) to which 100 U $ml^{-1}$ penicillin and 100 mg $ml^{-1}$ streptomycin had been added. The primary consideration was always to obtain a histopathological diagnosis and in cases of doubt, the bulk of the tumour was fixed for diagnostic use. Histologically, 15 tumours were classified as spindle cell, 17 as mixed and five as epithelioid tumours. The mitotic index varied from 0–12 mitoses $mm^{-2}$, with a median of 0.6 mitoses $mm^{-2}$. A further 10 tumours (nine choroidal, one ciliary body; five epithelioid, three spindle, two mixed type; mitotic index 0–2.6) were used in subsequent experiments to determine the effect of drug sequence in combination experiments. Approval for use of material not required for diagnosis was obtained from the Moorfields Eye Hospital Ethics Committee.

### ATP-based tumour chemosensitivity assay (ATP-TCA)

ATP-TCA was performed as previously described (Andreotti et al, 1995; Myatt et al, 1997). This assay is based on the linear relationship between ATP content and biomass (Petty et al, 1995). Cells were dissociated from melanoma tissue by incubation overnight at 37°C with 1.5 mg $ml^{-1}$ collagenase type H (Sigma Chemical Co Ltd., Poole, UK). The collagenase concentration was reduced to 0.75 mg $ml^{-1}$ from tumour 36 onwards. Following dissociation, the cells were washed in an antibiotic-containing serum-free complete assay medium (CAM, DCS Innovative Diagnostik Systeme, Hamburg, Germany) by centrifugation at 400 ***g*** for 10 min and their viability checked by trypan blue exclusion. Cell viability ranged from 50% to 95% (mean 85%). Ficoll-hypaque density centrifugation (Lymphoprep, Nycomed UK Ltd, Birmingham, UK) with two further washes was used in two cases to remove cell debris. The cells were adjusted to 200 000 viable cells $ml^{-1}$ in CAM and 100 μl added to the wells of a 96-well polypropylene microplate (Costar, High Wycombe, UK) to which doubling dilutions of four drugs in triplicate wells (in 100-μl volumes) had been added while the cells were being prepared. Test drug concentrations (TDCs) are based on pharmacokinetic data adjusted to provide good discrimination between tumours (Table 1) (Andreotti et al, 1995). Combinations used two to three drugs added simultaneously unless otherwise specified. All drugs were left in the plate for the duration of the culture period. One row was reserved for six control wells with 100 μl CAM only (MO) and six wells to which 100 μl of a maximum inhibitor of cell survival (MI, DCS Innovative Diagnostik Systeme) was added.

The plate was incubated for 6–7 days at 37°C with high humidity in 5% $CO_2$ and the cells observed every 2–3 days by microscopy to check for infection or overgrowth. At the end of the incubation period, ATP was extracted from the cells by addition of a detergent-based extractant (TCER, DCS Innovative Diagnostik Systeme) and 50 μl from each well was added to 3.5 ml polystyrene tubes (Sarstedt, Numbrecht, Germany) or white microtitre plates (Dynatech Ltd, Billinghurst, UK), for estimation of ATP levels by luminescence assay. The tubes were loaded into a Berthold LB953 luminometer (EG&G Berthold, Wildbad, Germany) set to inject 55 μl of luciferin–luciferase reagent (DCS Innovative Diagnostik Systeme). Light output expressed as relative light units (RLU) was used to determine the mean % inhibition of cell growth/survival in triplicate wells at each drug concentration according to the following equation: $1-((\text{Test}-\text{MI})/(\text{MO}-\text{MI}))\times 100$.

For subsequent sequence experiments, plates were made up with the first drug in 100-μl volumes down the plate as before and cells added in 50 μl rather than 100 μl to allow later addition of 50 μl of the second drug (final concentration = 50% TDC) at varying intervals. Cells were plated at 20 000 per well as before. Cultures were stopped, and their ATP content and the degree of inhibition were measured as usual. Six experiments were performed with cytosine arabinoside + treosulfan, and four with gemcitabine + treosulfan using primary uveal melanoma cells.

### Data analysis

The results of each assay were analysed individually in an Excel 5.0 spreadsheet (Microsoft) allowing graphical representation of the response (Figure 1) and collected in a database (Access 2.0, Microsoft). Wells responsible for high variation in MO or test results were examined and excluded as outliers or known errors (e.g. pipetting, excessive cell clumping). For comparison of responses between patients (Table 2), a simple logarithmic index was derived by summing the percentage inhibition at each level of TDC tested as Index = 700 – $\text{Sum}[\text{Inhibition}_{3,13\,\ldots\,200}]$ (Hunter et al, 1993). An arbitrary level of 50% inhibition (Index < 350), $IC_{50}$ and $IC_{90}$ were used to assess relative *ex vivo* sensitivity or resistance (Table 3). Combination effects were assessed for independent and combination effects using the method described by Poch et al (1990).

 © Cancer Research Campaign 1999

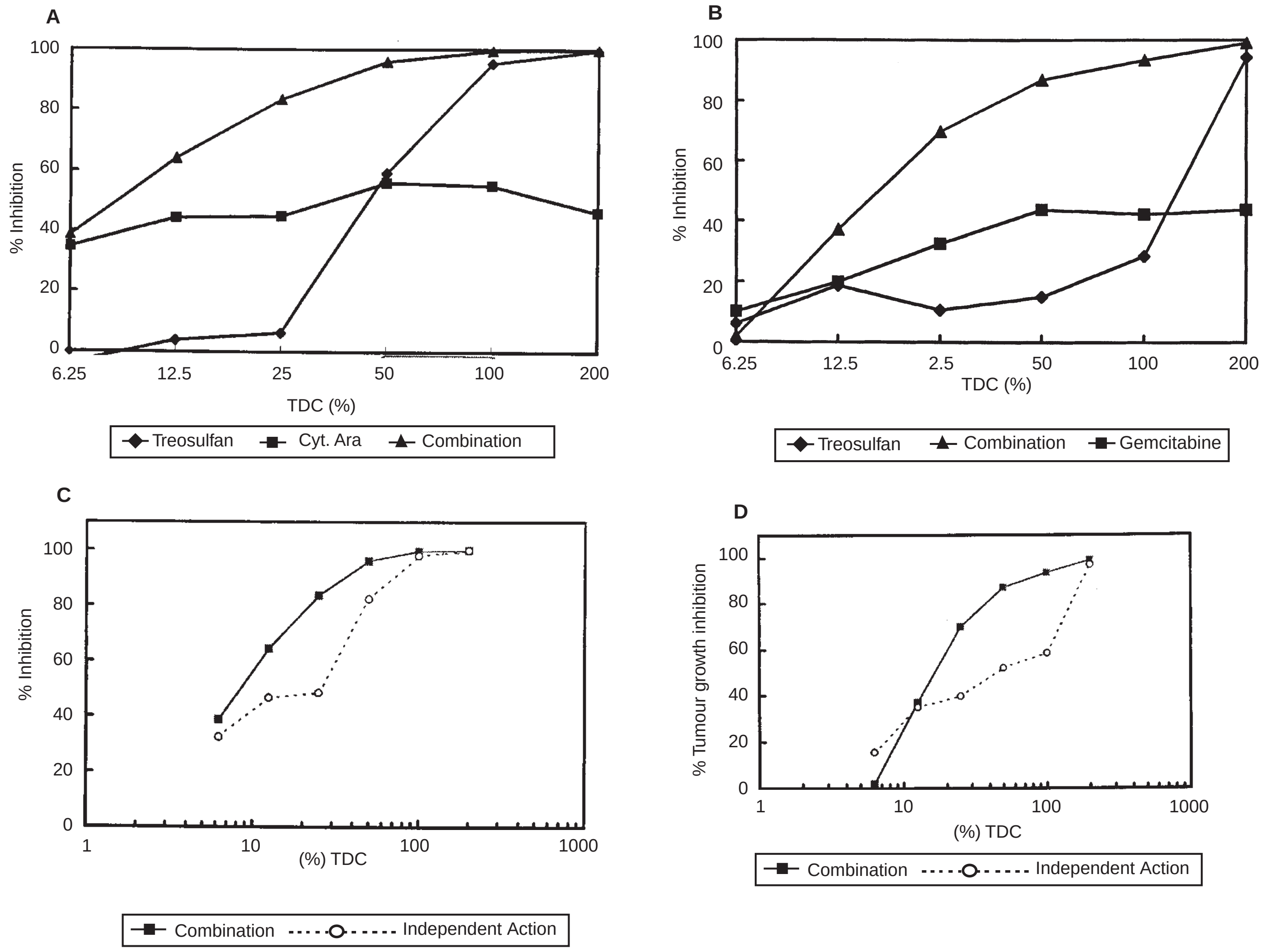

**Figure 1** Example ATP-TCA results for the combination of treosulfan with (**A**) cytosine arabinoside (case 15) and (**B**) gemcitabine (case 30) with combination effect graphs (Poch et al, 1990) for (**C**) treosulfan + cytosine arabinoside and (**D**) treosulfan + gemcitabine. In both sets of results, there is some sensitivity to both cytosine analogues at all concentrations tested, but this never reaches 100% inhibition. Treosulfan sensitivity is poor at most concentrations, but there is greater than 90% inhibition at high concentrations. However, in both cases, the combination is much more effective. TDC = test drug concentration

### RESULTS

Evaluable results were obtained from 31 of the 37 melanomas tested, giving an evaluability rate of 84%. Six tumours were non-evaluable: three had low MO values indicating death of most cells (without drug present) and three yielded too few cells after dissociation for assay (in one of these, the tumour was necrotic). No infections were encountered before or during culture.

The single agent results (TCA Index) are detailed in Table 3A, and the combination results in Table 3B to show the degree of heterogeneity in responsiveness to individual drugs/combinations between tumours. While most responded to treosulfan + cytosine analogue, these cases showed considerable variability in their response to other drugs. Table 2 gives a summary of the sensitivities using an arbitrary index of 350 as the cut-off below which the agent/combination was said to be sensitive, together with the number of cases in which the $IC_{50}$ and $IC_{90}$ fell within the range of concentrations tested. In this study, the treosulfan sensitivity was lower than previously observed (Myatt et al, 1997), although there was a dose response in most cases evidenced by the $IC_{50}$ and $IC_{90}$ values. Figure 1A shows a typical result from one tumour for the single agents and combination of treosulfan with cytosine arabinoside (Ara-C). This indicates modulation of the alkylating agent response by Ara-C, as observed previously (Myatt et al, 1997). Similar results were obtained with gemcitabine (Figure 1B), although gemcitabine alone exhibited a slightly more pronounced dose–response curve and showed less activity at low concentrations. The combination–response curves (Poch et al, 1990, 1995) suggest a greater effect of combining the two drugs than their independent actions would predict at most concentrations, particularly in the mid-range (Figure 1C and 1D).

While most tumours showed a response to treosulfan which could be modulated by cytosine analogues, there was considerable variation between individuals in the response to other single agents (Table 3). There was no relationship between sensitivity index for any of the drugs listed and mitotic rate, tumour size or cell type. Many tumours showed a response to cytosine arabinoside (43%) or gemcitabine (30%) as single agent. However, these drugs never induce 100% cell kill when present alone. Sensitivity to anthracyclines was observed in 7% of cases tested with doxorubicin and 11% tested with mitoxantrone. There was some indication of a lack of cross-resistance between these two

*© Cancer Research Campaign 1999* 

**Table 2** Summary of results using an arbitrary threshold for sensitivity of Index < 350, and the number of cases in which the $IC_{50}$ and $IC_{90}$ fell within the range of concentrations tested (3.13–200% TDC). Treosulfan is the most active alkylating agent tested and the combinations with cytosine arabinoside or gemcitabine are effective in most tumours tested. The median and range (in brackets) for each parameter in terms of TDC% are shown below, where these fall within the concentration range tested. na = not achieved.

| Drug | Sensitivity index | $IC_{50}$ | $IC_{90}$ |
|---|---|---|---|
| Cytosine arabinoside[a] | 12/28 (43%) | 19/29 (66%) | 1/29(3%) |
| | 387 (63–875) | 5 (4–60) | na |
| Doxorubicin | 2/27 (7%) | 10/23 (43%) | 0/23 (0%) |
| | 494 (307–1309) | 92 (9–159) | na |
| Treosulfan | 2/31 (6%) | 18/31 (58%) | 6/31 (19%) |
| | 491 (191–1003) | 105 (3–163) | 21 (14–53) |
| Vincristine/vinblastine | 0/14 (0%) | 0/14 (0%) | 0/14 (0%) |
| | 682 (482–1011) | na | na |
| Paclitaxel | 4/29 (14%) | 13/33 (39%) | 1/33 (3%) |
| | 525 (189–816) | 109 (3–186) | 199 |
| Gemcitabine | 6/20 (30%) | 8/20 (40%) | 0/20 (0%) |
| | 480 (237–918) | 85 (4–143) | na |
| Mitoxantrone | 1/9 (11%) | 5/9 (56%) | 0/9 (0%) |
| | 384 (258–514) | 112 (98–157) | na |
| Mitoxantrone + Paclitaxel | 3/9 (33%) | 9/9 (100%) | 3/9 (33%) |
| | 373 (258–485) | 134 (101–170) | 32 (22–40) |
| Doxorubicin + Paclitaxel | 8/26 (31%) | 20/26 (77%) | 9/26 (34%) |
| | 400 (233–657) | 125 (3–180) | 30 (22–39) |
| Treosulfan + Ara C | 25/29 (86%) | 27/29 (93%) | 20/29 (69%) |
| | 214 (28–611) | 3 (3–175) | 41 (6–121) |
| Treosulfan + Gemcitabine | 16/23 (70%) | 22/23 (96%) | 18/23 (78%) |
| | 306 (61–698) | 104 (3–161) | 28 (6–72) |
| Treosulfan + Doxorubicin | 1/8 (13%) | 7/8 (88%) | 3/8 (38%) |
| | 433 (178–625) | 107 (3–158) | 65 (28–73) |
| Treosulfan + Paclitaxel | 8/17 (47%) | 16/17 (94%) | 13/17 (76%) |
| | 353 (131–567) | 126 (3–175) | 32 (20–88) |
| Treosulfan + Paclitaxel + Ara C | 11/12 (92%) | 11/11 (100%) | 11/11 (100%) |
| | 148 (36–351) | 3 (3–134) | 43 (6–73) |
| Treosulfan + Paclitaxel + Gemcitabine | 9/11 (82%) | 11/11 (100%) | 10/11 (91%) |
| | 286 (189–456) | 105 (3–194) | 33 (24–43) |

[a]100% melanoma cell kill not achieved.

anthracyclines with generally greater sensitivity to mitoxantrone. Fourteen per cent of cases showed sensitivity to paclitaxel, but there were no responses to vinca alkaloids (vincristine or vinblastine). The combination of paclitaxel with doxorubicin showed activity in 31%, while mitoxantrone + paclitaxel was effective in 33%.

Treosulfan + cytosine arabinoside was the best of the double-agent combinations, with 86% of patients showing sensitivity. The combination of treosulfan with gemcitabine showed similar efficacy in 70% cases. While the highest sensitivity was seen for the combination of treosulfan + paclitaxel + cytosine arabinoside (92%), in general paclitaxel added little to the sensitivity of the combination (Figure 2A). However, in occasional patients with limited treosulfan sensitivity and some paclitaxel sensitivity, paclitaxel addition does improve the response (Figure 2B).

Schedule experiments combining treosulfan at different times with cytosine arabinoside or gemcitabine are shown in Figure 3. These experiments were conducted with 50% TDC concentrations of cytosine arabinoside or gemcitabine and show simultaneous addition to be most effective. Prior addition of gemcitabine or cytosine arabinoside before treosulfan abrogated the effect of the alkylating agent and in this sequence the combination failed to produce 100% inhibition.

### DISCUSSION

In the UK, rare tumours (i.e. those outside the top 10 for incidence) account for 25% of deaths from cancer (Thames Cancer Registry, 1995). Choroidal melanoma is rare with an age-standardized incidence of 0.4 to 1.2 cases per 100 000 within Europe (Foss and Dolin, 1996). Consequently, clinical trials are difficult and rarely performed as funding bodies, whether industrial or charitable, prefer to concentrate resources on more common tumours. Yet these patients need treatment. Chemosensitivity testing has a poor reputation following a long history of technical problems. However modern methods offer a way of testing new drugs and regimens in rare tumours which could not be contemplated in the clinical setting. ATP-based methods have theoretical advantages of sensitivity and reproducibility over other methods (Petty et al, 1995; Cree and Kurbacher, 1997). The ATP-TCA overcomes most of the difficulties which have beset chemosensitivity testing and shows considerable promise as a way of individualizing chemotherapy (Cree and Kurbacher, 1997). Correlation of sensitivity in the ATP-TCA with clinical response is comparable with bacteriological or oestrogen receptor testing at 75–80% (Andreotti et al, 1995; Cree et al, 1996; Cree and Kurbacher, 1997). For choroidal melanoma, our evaluability rate has improved from 71%

 *© Cancer Research Campaign 1999*

**Table 3** Results of testing for each tumour expressed as a simple summary index of inhibition across the range of concentrations tested for (**A**) single agents and (**B**) Paclitaxel and combinations. Low values indicate considerable inhibition, while higher values indicate resistance. Values greater than 700 indicate growth greater than control wells, which is likely to be artefactual and simply reflects resistance. nd = not done.

**(A)**

| Number | Cytosine arabinoside | Doxorubicin | Treosulfan Vinblastine[a] | Vincristine | Paclitaxel | Gemcitabine | Mitoxantrone |
|---|---|---|---|---|---|---|---|
| 1 | 685 | 465 | 539 | 671 | 465 | nd | nd |
| 3 | 641 | 429 | 510 | 682 | 220 | nd | nd |
| 4 | 752 | 606 | 720 | 604 | 393 | nd | nd |
| 5 | 820 | 307 | 739 | 725 | 407 | nd | nd |
| 7 | 248 | 449 | 380 | nd | 410 | nd | nd |
| 8 | 653 | 539 | 566 | 482 | 525 | nd | nd |
| 9 | 274 | 633 | 402 | nd | 476 | nd | nd |
| 11 | 181 | 320 | 600 | 592 | 189 | 273 | nd |
| 12 | 63 | 453 | 491 | nd | 464 | 237 | nd |
| 13 | 196 | 501 | 507 | 759 | 500 | 284 | nd |
| 14 | 180 | 337 | 462 | nd | 478 | nd | nd |
| 15 | 367 | 541 | 420 | nd | 556 | nd | nd |
| 16 | 423 | 615 | 364 | 633 | 580 | 529 | nd |
| 17 | 670 | 464 | 623 | 613 | 581 | 605 | nd |
| 18 | 183 | 382 | 191 | nd | 582 | nd | nd |
| 19 | 379 | 513 | 449 | 767 | 329 | 587 | nd |
| 20 | 453 | 638 | 521 | 891 | 816 | 918 | nd |
| 21 | 480 | nd | 397 | nd | 327 | nd | nd |
| 23 | 396 | 409 | 456 | 726 | 569 | 589 | nd |
| 24 | 627 | 781 | 1003 | 1011 | nd | 678 | nd |
| 26 | nd | nd | 389 | nd | 607 | 460 | nd |
| 27 | 498 | 654 | 472 | nd | 653 | 501 | 384 |
| 28 | nd | nd | 345 | nd | 530 | 429 | 339 |
| 29 | 320 | 494 | 446 | nd | 611 | 638 | 442 |
| 30 | 601 | 475 | 520 | nd | 594 | 515 | 361 |
| 31 | 173 | 529 | 400 | nd | 442 | 328 | 373 |
| 33 | 331 | 611 | 522 | nd | 541 | 456 | 258 |
| 34 | nd | nd | 633 | nd | nd | 330 | nd |
| 35 | 875 | 1309 | 520 | nd | 677 | 576 | 485 |
| 36 | 261 | 476 | 573 | nd | 640 | 414 | 406 |
| 37 | 311 | 483 | 429 | nd | 439 | 324 | 514 |

[a]Vinblastine used from 97M011 onwards.

**(B)**

| Number | Mitoxantrone + Paclitaxel | Doxorubicin + Pacltaxel | Treosulfan + Ara C | Treosulfan + Gemcitabine | Treosulfan + Doxorubicin | Treosulfan + Paclitaxel | Treosulfan + Paclitaxel + Ara C | Treosulfan + Paclitaxel + Gemcitabine |
|---|---|---|---|---|---|---|---|---|
| 1 | nd | nd | nd | nd | nd | nd | nd | nd |
| 3 | nd | 334 | 250 | nd | nd | nd | nd | nd |
| 4 | nd | 357 | 464 | nd | nd | nd | nd | nd |
| 5 | nd | 284 | 611 | nd | nd | nd | nd | nd |
| 7 | nd | 462 | 206 | nd | nd | nd | nd | nd |
| 8 | nd | 403 | 582 | nd | nd | nd | nd | nd |
| 9 | nd | 347 | 148 | nd | nd | nd | nd | nd |
| 11 | nd | 233 | 73 | 210 | 178 | 131 | 119 | nd |
| 12 | nd | 399 | 28 | 61 | 461 | 375 | 36 | nd |
| 13 | nd | 401 | 99 | 100 | 625 | 302 | 68 | nd |
| 14 | nd | 293 | 90 | 130 | nd | nd | 54 | nd |
| 15 | nd | 513 | 191 | 228 | nd | nd | 173 | nd |
| 16 | nd | 483 | 242 | 387 | 543 | 334 | 242 | nd |
| 17 | nd | 424 | 513 | 374 | 406 | 368 | 302 | nd |
| 18 | nd | 441 | 98 | 86 | nd | nd | 123 | nd |
| 19 | nd | 283 | 214 | 315 | 406 | 202 | 257 | nd |
| 20 | nd | 657 | 312 | 378 | 496 | 567 | 351 | nd |
| 21 | nd | nd | 203 | nd | nd | nd | 118 | nd |
| 23 | nd | 367 | 257 | 431 | 391 | 291 | 180 | nd |
| 24 | nd | nd | 303 | 698 | nd | nd | nd | nd |
| 26 | nd | nd | 260 | 367 | nd | nd | nd | 402 |
| 27 | 384 | 640 | 275 | 343 | nd | 555 | nd | 319 |
| 28 | 339 | 487 | 125 | 237 | nd | 353 | nd | 249 |
| 29 | 442 | 459 | 140 | 218 | nd | 392 | nd | 198 |
| 30 | 361 | 400 | 276 | 312 | nd | 461 | nd | 286 |
| 31 | 373 | 311 | 113 | 273 | nd | 284 | nd | 189 |
| 33 | 258 | 304 | 160 | 339 | nd | 336 | nd | 336 |
| 34 | nd | nd | nd | 245 | nd | nd | nd | 228 |
| 35 | 485 | 565 | 281 | 389 | nd | 463 | nd | 456 |
| 36 | 337 | 514 | 251 | 306 | nd | 470 | nd | 311 |
| 37 | 426 | 263 | 129 | 254 | nd | 222 | nd | 221 |

*© Cancer Research Campaign 1999* 

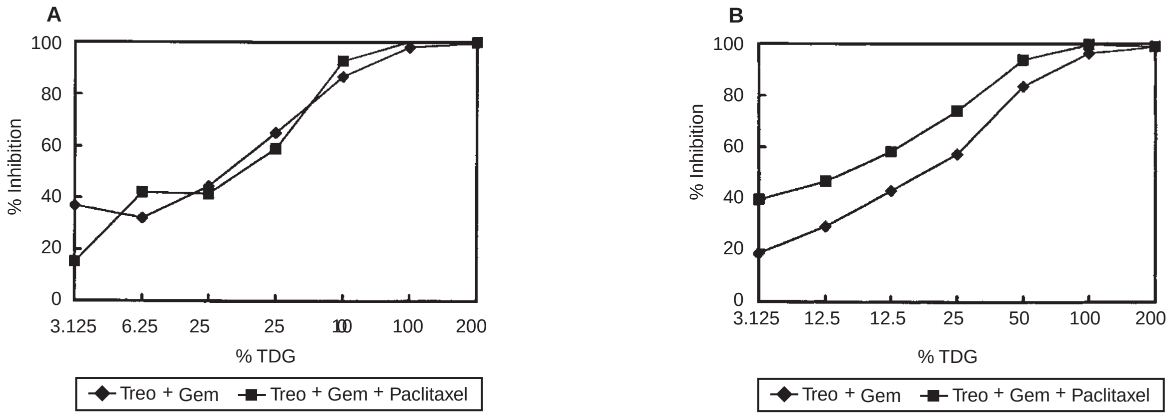

**Figure 2** Examples showing addition of paclitaxel to the combination of treosulfan + gemcitabine. In case 28 (**A**) there is no effect, while in case 31 (**B**) a small effect is observed. TDC = test drug concentration

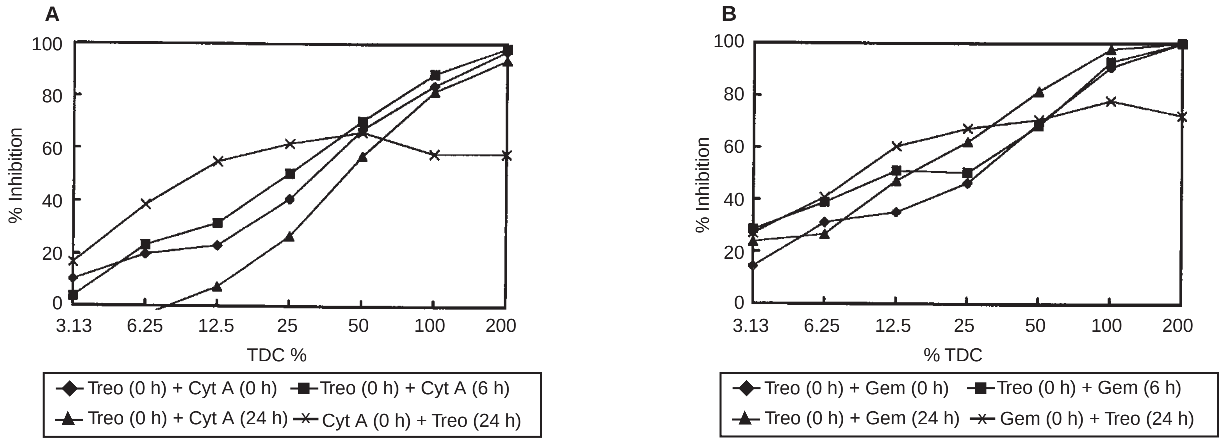

**Figure 3** Sequential studies with (**A**) cytosine arabinoside and (**B**) gemcitabine in combination with treosulfan. When either cytosine analogue is given before the treosulfan, the combination fails to acheive 100% inhibition. In both cases, best inhibition is achieved by concomitant administration of the two drugs. TDC = test drug concentration in the first 28 cases tested (Myatt et al, 1997) to 86% in this series (the current series includes four of the first series tumours in which combinations were tested). The improvement probably reflects the gentler dissociation and cell handling used in comparison with our initial practice (Myatt et al, 1997).

Our previous (Myatt et al, 1997) and current results confirm the chemoresistance of choioidal melanoma, but also suggest that there are options for improvement of response rates. The current study shows few differences from our previous study. Although the sensitivity of the melanomas to treosulfan was reduced (Table 2), examination of the individual data shows that most tumours (Table 3) did exhibit some sensitivity to this agent, which remains the best of the alkylating agents we have tried in the assay. We have previously shown that these tumours are relatively unresponsive to temozolomide (Myatt et al, 1997), a drug closely related to dacarbazine (DTIC) which forms the mainstay of treatment for cutaneous melanoma, but has poor results against uveal melanoma (Bedikian et al, 1995). Results for other agents were similar to those obtained previously, with confirmation of some sensitivity of uveal melanoma to paclitaxel, cytosine arabinoside and anthracyclines. As before, vinca alkaloids showed little effect.

There is heterogeneity of chemosensitivity (Table 2), as we have previously observed in many tumour types (Hunter et al, 1993; Andreotti et al, 1995), but on the basis of these results many previously untreated uveal melanomas are likely to respond to the combination of treosulfan with cytosine analogues. However, it should be noted that we have tested primary tumour material, not metastases, and it is possible that these may show differences in chemosensitivity. For clinical use, we have chosen to pursue the combination of gemcitabine + treosulfan, as the pharmacokinetics and activity of gemcitabine in solid tumours are superior to cytosine arabinoside. Preliminary clinical use of this combination in 18 heavily pre-treated patients with recurrent ovarian or breast carcinoma based on sensitivity in ATP-TCA is encouraging (data not shown) with a good safety profile and we believe that it may have wider applicability. A phase I/II trial of treosulfan + gemcitabine in metastatic uveal melanoma is now in progress, and we have commenced a phase II trial of assay-directed chemotherapy in melanoma patients with accessible disease, based on this data and studies of skin melanoma (Neale et al, unpublished data).

The differences we have observed between gemcitabine and cytosine arabinoside are interesting. Although these agents show

 *© Cancer Research Campaign 1999*

close cross-sensitivity, and both are cytosine analogues, there are major differences in their pharmacology (Peters et al, 1996). Several biochemical explanations are possible, but there is little doubt that gemcitabine is a useful drug in combination with alkylating agents in particular (Peters et al, 1996; Iwasaki et al, 1997; Bruckner HW, personal communication). The explanation for the apparent modulation of treosulfan sensitivity observed with both drugs may be due to inhibition of DNA repair of alkylating agent-induced cross-links, to direct incorporation of the analogues into DNA, to changes in dNTP pools (Peters et al, 1996; Iwasaki et al, 1997), or to a combination of several of these mechanisms. Further studies using assays of DNA repair and measurement of dNTP pools are required, but our results show clear evidence of synergy between the alkylating agent and cytosine analogue in primary uveal melanoma for both drugs given simultaneously.

Analysis of the single agent responses confirms our previous data showing a lack of activity of vinca alkaloids in this tumour, but some activity for paclitaxel. We had hoped that addition of paclitaxel to the combination of treosulfan with cytosine analogues might further improve responses, but in general the data do not support addition of this agent which would also add considerable toxicity. Occasional patients respond better to paclitaxel + anthracyclines than treosulfan + cytosine analogues. This was observed in three of the early cases studied (Table 3B) and it is clear from our data that there may be a role for paclitaxel and anthracyclines in patients refractory to treosulfan + cytosine analogue. Routine chemosensitivity testing to individualize testing is probably not appropriate in these patients, though it is certainly feasible, and current phase III trials of the technology may alter this view.

In this study, the ATP-TCA has shown itself able to predict combinations suitable for use in rare solid tumours in which there is no prospect of doing the number of phase II trials which would be required using current empirical methods. As the number of agents available continues to grow, the need for some form of preclinical planning of phase II trials becomes more apparent. The method used here allows large numbers of mechanistically logical permutations to be tested with material from small numbers of patients with potential benefits in terms of development time and expense.

### ACKNOWLEDGEMENTS

We wish to thank Dr P Ashman (Schering-Plough Ltd), Ms Marylin Shedden (medac Ltd), and Dr Alison Jeynes (Bristol-Myers Ltd) for their assistance in providing stock drugs used in the assay. We are grateful to Mr R Alexander, Ms R Hart and Ms R Patel for their assistance and to Dr G J Peters for his advice. This research was funded by the Guide Dogs for the Blind Association and a donation from Schering-Plough Ltd.

### REFERENCES

Albert DM, Niffenegger AS and Willson JK (1992) Treatment of metastatic uveal melanoma: a review and recommendations. *Surv Ophthalmol* **36**: 429–438

Albert DM, Ryan LM and Borden EC (1996) Metastatic ocular and cutaneous melanoma: a comparison of patient characteristics and prognosis. *Arch Ophthalmol* **114**: 107–108

Andreotti PE, Cree IA, Kurbacher CM, Hartmann DM, Linder D, Harel G, Gleiberman I, Caruso PA, Ricks SH, Untch M, Sartori C and Bruckner HW (1995) Chemosensitivity testing of human tumors using a microplate adenosine triphosphate luminescence assay: clinical correlation for cisplatin resistance of ovarian carcinoma. *Cancer Res* **55**: 5276–5282

Bedikian AY, Legha SS, Mavligit G, Carrasco CH, Khorana S, Plager C, Papadopoulos N and Benjamin RS (1995) Treatment of uveal melanoma metastatic to the liver: a review of the M.D. Anderson Cancer Center experience and prognostic factors. *Cancer* **76**: 1665–1670

Cantore M, Fiorentini G, Aitini E, Davitti B, Cavazzini G, Rabbi C, Lusenti A, Bertani M, Morandi C and Benedini V (1994) Intra-arterial hepatic carboplatin-based chemotherapy for ocular melanoma metastatic to the liver. Report of a phase II study. *Tumori* **80**: 37–39

Chana JS, Cree IA, Foss AJE, Hungerford JL and Wilson GD (1998) The prognostic significance of c-myc oncogene expression in uveal melanoma. *Melanoma Res* **8**: 139–144

Cree IA, Kurbacher CM, Untch M, Sutherland LA, Hunter EMM, Subedi AMC, James EA, Dewar JA, Preece PE, Andreotti PE and Bruckner HW (1996) Correlation of the clinical response to chemotherapy in breast cancer with ex vivo chemosensitivity. *Anti-Cancer Drugs* **7**: 630–635

Cree IA and Kurbacher CM (1997) Individualising chemotherapy for solid tumours – is there any alternative? *Anti-Cancer Drugs* **8**: 541–548

Foss AJ and Dolin PJ (1996) Trends in eye cancer mortality among adults in the USA and England and Wales. *Br J Cancer* **74**: 1687–1689

Foss AJE, Alexander RA, Jefferies LW, Hungerford JL, Harris AL and Lightman S (1996) Microvessel count predicts survival in uveal melanoma. *Cancer Res* **56**: 2900–2903

Hunter EM, Sutherland LA, Cree IA, Dewar JA, Preece PE and Andreotti PE (1993) Heterogeneity of chemosensitivity in human breast carcinoma: use of an adenosine triphosphate (ATP) chemiluminescence assay. *Eur J Surg Oncol* **19**: 242–249

Iwasaki H, Huang P, Keating J and Plunkett W (1997) Differential incorporation of Ara-C, gemcitabine and fludarabine into replicating and repairing DNA in proliferating human leukaemia cells. *Blood* **90**: 270–278

Kurbacher CM, Bruckner HW, Cree IA, Kurbacher JA, Wilhelm L, Poch G, Indefrei D, Mallman P, Krebs D and Andreotti PE (1997) Mitoxantrone combined with paclitaxel as salvage therapy for platinum-refractory ovarian cancer: laboratory study and clinical pilot trial. *Clin Cancer Res* **3**: 1527–1533

Kurbacher CM, Cree IA, Bruckner HW, Mallmann P and Andreotti PE (1998) Chemotherapy directed by the ATP tumour chemosensitivity assay improves response rates and survival for patients with recurrent ovarian cancer. *Anti-Cancer Drugs* **9**: 51–57

Myatt N, Cree IA, Kurbacher CM, Foss AJE, Hungerford JL and Plowman PN (1997) The ex vivo chemosensitivity profile of choroidal melanoma. *Anti-Cancer Drugs* **8**: 756–762

Peters GJ, Ruiz van Haperen VW, Bergman AM, Veerman G, Smitskamp-Wilms E, van Moorsel CJ, Kuiper CM and Braakhuis BJ (1996) Preclinical combination therapy with gemcitabine and mechanisms of resistance. *Semin Oncol* **23** (Suppl 10): 16–24

Petty RD, Sutherland LA, Hunter EM and Cree IA (1995) Comparison of MTT and ATP-based assays for the measurement of viable cell number. *J Biolumin Chemilumin* **10**: 29–34

Plunkett W, Huang P and Gandhi V (1995) Preclinical characteristics of gemcitabine. *Anticancer Drugs* **6** (Suppl 6): 7–13

Poch G, Reiffenstein RJ and Unkelbach HD (1990) Application of the isobologram technique for the analysis of combined effects with respect to additivity as well as independence. *Can J Physiol Pharmacol* **68**: 682–688

Poch G, Reiffenstein RJ and Baer HP (1995) Quantitative estimation of potentiation and antagonism by dose ratios corrected for slopes of dose–response curves deviating from one. *J Pharmacol Toxicol Methods* **33**: 197–204

Proebstle TM, Scheibenbogen C, Sterry W and Keilholz U (1996) A phase II study of dacarbazine, cisplatin, interferon-alpha and high-dose interleukin-2 in 'poor-risk' metastatic melanoma. *Eur J Cancer* **32A**: 1530–1533

Raivio I (1977) Uveal melanoma in Finland. An epidemiological, clinical, histological and prognostic study. *Acta Ophthalmol* **133**(Suppl): 1–64

Thames Cancer Registry (1995) *Cancer in south-east England 1992*

van Moorsel CJ, Veerman G, Bergman AM, Guechev A, Vermorken JB, Postmus PE and Peters GJ (1997) Combination chemotherapy studies with gemcitabine. *Semin Oncol* **24** (Suppl 7): S7.17–S7.23

© Cancer Research Campaign 1999 
